# Prediction of Response to Systemic Corticosteroids in Active UC by Microbial Composition—A Prospective Multicenter Study

**DOI:** 10.1093/ibd/izad126

**Published:** 2023-07-18

**Authors:** Andreas Blesl, Philipp Wurm, Silvio Waschina, Hans Peter Gröchenig, Gottfried Novacek, Christian Primas, Walter Reinisch, Maximilian Kutschera, Constanze Illiasch, Barbara Hennlich, Pius Steiner, Robert Koch, Wolfgang Tillinger, Thomas Haas, Gerhard Reicht, Andreas Mayer, Othmar Ludwiczek, Wolfgang Miehsler, Karin Steidl, Lukas Binder, Simon Reider, Christina Watschinger, Stefan Fürst, Patrizia Kump, Alexander Moschen, Konrad Aden, Gregor Gorkiewicz, Christoph Högenauer

**Affiliations:** Department of Internal Medicine, Division of Gastroenterology and Hepatology, Medical University of Graz, Graz, Austria; Department of Internal Medicine, Division of Gastroenterology and Hepatology, Medical University of Graz, Graz, Austria; Institute of Pathology, Medical University of Graz, Graz, Austria; Christian-Albrechts-University Kiel, Institute for Human Nutrition and Food Science, Nutriinformatics, Kiel, Germany; Brothers of Saint John of God Hospital, St. Veit an der Glan, Austria; Department of Internal Medicine III, Division of Gastroenterology and Hepatology, Medical University of Vienna, Vienna, Austria; Department of Internal Medicine III, Division of Gastroenterology and Hepatology, Medical University of Vienna, Vienna, Austria; Department of Internal Medicine III, Division of Gastroenterology and Hepatology, Medical University of Vienna, Vienna, Austria; Department of Internal Medicine III, Division of Gastroenterology and Hepatology, Medical University of Vienna, Vienna, Austria; Hospital Landstraße, Vienna, Austria; Hospital Landstraße, Vienna, Austria; Hospital Wels-Grieskirchen, Wels, Austria; Medical University of Innsbruck, Innsbruck, Austria; Franziskus Hospital, Vienna, Austria; Darmpraxis, Salzburg, Austria; Brothers of Saint John of God Hospital, Graz, Austria; University Hospital, St.Pölten, Austria; Hospital Hall, Hall, Austria; Brothers of Saint John of God Hospital, Salzburg, Austria; Brothers of Saint John of God Hospital, St. Veit an der Glan, Austria; Department of Internal Medicine, Division of Gastroenterology and Hepatology, Medical University of Graz, Graz, Austria; Department of Internal Medicine 2 (Gastroenterology and Hepatology), Faculty of Medicine, Kepler University Hospital, Johannes Kepler University, Linz, Austria; Christian Doppler Laboratory for Mucosal Immunology, Johannes Kepler University Linz, Linz, Austria; Department of Internal Medicine 2 (Gastroenterology and Hepatology), Faculty of Medicine, Kepler University Hospital, Johannes Kepler University, Linz, Austria; Christian Doppler Laboratory for Mucosal Immunology, Johannes Kepler University Linz, Linz, Austria; Department of Internal Medicine, Division of Gastroenterology and Hepatology, Medical University of Graz, Graz, Austria; Department of Internal Medicine, Division of Gastroenterology and Hepatology, Medical University of Graz, Graz, Austria; Department of Internal Medicine 2 (Gastroenterology and Hepatology), Faculty of Medicine, Kepler University Hospital, Johannes Kepler University, Linz, Austria; Christian Doppler Laboratory for Mucosal Immunology, Johannes Kepler University Linz, Linz, Austria; Institute of Clinical Molecular Biology, Christian-Albrechts-University and University Hospital Schleswig-Holstein, Kiel, Germany; Department of Internal Medicine I, Christian-Albrechts-University and University Hospital Schleswig-Holstein, Kiel, Germany; Institute of Pathology, Medical University of Graz, Graz, Austria; Department of Internal Medicine, Division of Gastroenterology and Hepatology, Medical University of Graz, Graz, Austria

**Keywords:** ulcerative colitis, microbiome, butyrate, corticosteroids

## Abstract

**Background:**

Corticosteroids are used for induction of remission in patients with moderately to severely active ulcerative colitis. However, up to one-third of patients fail to this therapy. We investigated if fecal microbial composition or its metabolic capacity are associated with response to systemic corticosteroids.

**Methods:**

In this prospective, multicenter study, patients with active ulcerative colitis (Lichtiger score ≥4) receiving systemic corticosteroids were eligible. Data were assessed and fecal samples collected before and after 4 weeks of treatment. Patients were divided into responders (decrease of Lichtiger Score ≥50%) and nonresponders. The fecal microbiome was assessed by the 16S rRNA gene marker and analyzed with QIIME 2. Microbial metabolic pathways were predicted using parsimonious flux balance analysis.

**Results:**

Among 93 included patients, 69 (74%) patients responded to corticosteroids after 4 weeks. At baseline, responders could not be distinguished from nonresponders by microbial diversity and composition, except for a subgroup of biologic-naïve patients. Within 4 weeks of treatment, responders experienced changes in beta diversity with enrichment of ascribed beneficial taxa, including *Blautia, Anaerostipes,* and *Bifidobacterium*, as well as an increase in predicted butyrate synthesis. Nonresponders had only minor longitudinal taxonomic changes with a significant increase of *Streptococcus salivarius* and a microbial composition shifting away from responders.

**Conclusion:**

Baseline microbial diversity and composition seem to be of limited use to predict response to systemic corticosteroids in active ulcerative colitis. Response is longitudinally associated with restoration of microbial composition and its metabolic capacity.

## Teaser

In this prospective study, patients with active ulcerative colitis receiving systemic corticosteroids were included (*n*  = 93). Baseline microbial diversity and composition were associated with response to treatment only in biologic-naïve patients. Responders experienced longitudinal restoration of microbial composition and function.

**Key Messages**

**What is already known?**

Inflammatory bowel disease patients have perturbations of the fecal microbiome, and the microbial composition has been reported to enable prediction of response to certain biologics.

**What is new here?**

The microbial diversity and composition before treatment were associated with response to systemic corticosteroids in active ulcerative colitis only in the subgroup of biologic-naïve patients. Response to treatment led to longitudinal restoration of the microbial composition and metabolic capacity.

**How can this study help patient care?**

Likely, other factors than the baseline microbial composition are primarily responsible for the efficacy of corticosteroids in active ulcerative colitis and need to be determined and considered in treatment decisions.

## Introduction

Ulcerative colitis (UC) is an inflammatory bowel disease (IBD) affecting the colonic mucosa. The disease behavior is characterized by varying inflammation burden.^1^ Despite the availability of modern biologics, corticosteroids are still recommended for induction of remission in patients with moderately to severely active UC.^2^ Up to one-third of UC patients treated with corticosteroids do not respond, and predictors of response are still lacking.^3,4^ Likewise, little is known about changes in microbial composition and metabolic pathways during corticosteroid treatment. Thus, a microbiome signature indicating UC patients refractory to corticosteroids would be very useful for clinical practice to avoid inefficient exposures associated with potential side effects.

Previous investigations in IBD patients suggested that microbial composition or its metabolic capacity might predict treatment response to biologics or immunosuppressants.^5–9^ Microbial butyrate synthesis has been shown to be predictive for treatment efficacy of azathioprine in IBD patients.^5^ Further, response to antitumor necrosis factor (anti-TNF) treatment in UC has been predicted by differential expression of antimicrobial peptides, lower dysbiosis indices, and increased abundance of *F. prausnitzii* in mucosal biopsies before treatment initiation.^6^ Efficacy of vedolizumab (UC and Crohn’s disease) and ustekinumab (Crohn’s disease) has also been associated with microbial diversity, composition, and function.^7,8^

We conducted an investigator-initiated, prospective, multicenter study including patients with active UC being treated with systemic corticosteroids. The primary aim of this study was to investigate if response to corticosteroids could be predicted by fecal microbial composition or function before treatment initiation. In addition, we aimed to study longitudinal changes in the fecal microbiome and its metabolic function in relation to treatment response.

## Methods

### Study Population

Between May 2018 and December 2020, patients with a diagnosis of UC at 18 study centers in Austria were eligible if suffering from active disease and being scheduled for treatment with oral or intravenous systemic corticosteroids (prednisolone or methylprednisolone). Active disease was defined by a Lichtiger Score ≥4. The Lichtiger score is determined by 8 variables: diarrhea, nocturnal stools, visible blood in stool, fecal incontinence, abdominal pain/cramping, general well-being, abdominal tenderness, and need for antidiarrheals. The Lichtiger score ranges from 0 (no activity) to 21 points (maximal activity).^10^ Severe colitis was defined as Lichtiger Score >10.^11^ Exclusion criteria were bacterial, viral, or parasitic infections and diagnosis of Crohn’s disease. Concomitant medications to treat UC were allowed in a stable dose if the treatment was started before initiation of corticosteroids. The start of probiotics and biologics or small molecules during the active study period led to exclusion from the analysis. Corticosteroid dosing and tapering were performed in accordance with the clinical practice at each study center.

The study was approved by the research ethics committees at the Medical University of Graz (EK 29-316 ex 16/17) and authorized by local ethics committees for each participating study center. It was registered at clinicaltrials.gov (NCT03460847) and was performed in accordance with the ethical standards laid down in the Declaration of Helsinki and its amendments. Written informed consent was obtained from all patients.

### Data Collection

Patient characteristics and clinical scores were assessed, and fecal samples collected at time of corticosteroid initiation (V1) and after 4 weeks of treatment (V2).

### End Points

The primary objective of this study was to determine predictive differences in microbial composition and function between week 4 responders and nonresponders to corticosteroids prior to treatment. In addition, we investigated longitudinal changes in microbial diversity, composition, and metabolic function in association with treatment response. Response to corticosteroid treatment was defined as decrease of the Lichtiger Score ≥50% from V1 to V2.^10^

### Statistical Analysis

Patient characteristics were reported as absolute and relative frequencies for categorical data and numerical data as medians and interquartile ranges (q1, q3). Comparisons between groups were done using *t* tests, Mann-Whitney *U* tests, or χ^2^ tests as appropriate. SPSS Version 26 was used for statistical calculations and Graph Pad Prism Version 9 for illustration.

### Calprotectin/Lipocalin-2

Fecal calprotectin and fecal lipocalin-2 (LCN-2) were assessed in a central lab. Native stool samples were stored at -80°C before analysis. For calprotectin, the S100A8/S100A9 heterodimer DuoSet ELISA (R&D systems, Minneapolis) was used, and for LCN2, the human Lipocalin-2/NGAL DuoSet ELISA (R&D systems, Minneapolis) was used, both according to the manufacturer’s instructions. Individual dilutions were performed for all samples outside the assays’ standard curves to receive absolute numbers.

### Microbiome Analysis

#### DNA isolation and 16S amplicon sequencing

Fecal samples were collected from the patients at home or at the local study centers into special collection tubes with DNA stabilizer (Stratec molecular, Berlin, Germany) and were sent to the central study center (Medical University of Graz) by regular mail. The samples were then stored in the same tubes according to the manufacturer’s instructions until DNA extraction. Total fecal DNA was isolated with a combination of mechanical and enzymatic lysis using the MagNA Pure LC DNA Isolation Kit III (Bacteria, Fungi; Roche, Cat. No. 03264785001) according to manufacturer’s instructions. For this purpose, 250 µL of a prediluted sample (~1:1 dilution in 1x phosphate buffered saline (PBS); VWR, Cat. No. K812) and 250 µL of bacteria lysis buffer were transferred into MagNa Lyser Green Beads tubes (Roche, Cat. No. 03358941001); 1xPBS served as a negative control for pre-isolation steps and automated DNA isolation. Mechanical lysis was done using the MagNA Lyser Instrument (Roche) at 6500 rpm for 30 seconds 2 times. The samples were then incubated with 25 µL of lysozyme (Roche, Cat. No. 10837059001; dissolved to 100 mg/mL in 5 % glycerol/PBS) at 37°C for 30 minutes and with 43.4 µL of Proteinase K at 65°C for 60 minutes. After inactivation of enzymes at 95°C for 10 minutes, the MagNA Pure LC Instrument (Roche) was used to perform DNA isolation from 100 µL of each sample. Total DNA was eluted in 100 µL of elution buffer and stored at 20°C until 16S polymerase chain reaction (PCR).

For PCR amplification of hypervariable 16S rRNA gene regions, the primers 515F (GTGYCAGCMGCCGCGGTAA) and 806R (GGACTACNVGGGTWTCTAAT), synthesized at Eurofins, were used. The PCR reaction was performed in triplicates. A 25 µL of reaction setup contained 2 µL of total DNA extract, PCR-grade water (VWR Chemicals, Cat. No. E476), 1x fast start high fidelity buffer, 200 µM of deoxynucleotide triphosphate (dNTPs), 1.25 U high fidelity enzyme [5 U/µL] (FastStart High Fidelity PCR System dNTPack from Roche, Cat. No. 4738292001) and 0.4 µM of each 16S primer. Polymerase chain reaction cycling conditions were 95°C for 3 minutes; 30 cycles of 95°C for 45 seconds, 55°C for 45 seconds, and 72°C for 60 seconds; and 72°C for 7 minutes.

After amplification, technical triplicates were pooled, and amplification verification was done by agarose gel electrophoresis; 25 µL of PCR product were normalized according to manufacturer’s instructions on a SequalPrep Normalization Plate (ThermoFisher Scientific, Cat. No. A1051001).

Indexing PCR was performed using the same reaction setup and cycling conditions as described for the targeted PCR. Exceptions were the DNA template (7.5 µL of normalized PCR product) and the number of cycles (8 times). Barcoded primers were synthesized at Eurofins.

After index PCR, 5 µL of each sample was pooled and purified using gel electrophoresis and the Qiaquick Gel Extraction Kit (Qiagen, Cat. No. 28704) according to manufacturer’s instructions. The library then was quantified and sequenced in an Illumina Miseq Sequencer using v3 600 cycles chemistry.

#### Computational methods

For analysis of 16S amplicons, sequences were imported to Qiime2 (v. 2021.2).^12^ Quality control was performed using DADA2 (denoise-paired)^13^ to improve the overall sequence quality. Primers were trimmed and forward reads were truncated at nucleotide 220 and reverse reads at nucleotide 150, leading to total frequency of 6 310 277 in 191 samples with 2790 different features and a mean frequency of 33 038.1 per sample (Minimum frequency: 14 853; Maximum frequency: 65 048). To assign taxonomy to our 16S sequences, we used a pretrained Naïve Bayes Classifier that was trained on Silva 138 99% operational taxonomic units (OTUs),^14^ where sequences have been trimmed to include only sequences from the V4 region that was used for 16S amplicon sequencing according to the Earth Microbiome Project.^15^ Mitochondria and chloroplast reads were subsequently excluded from feature tables.

After quality filtering samples had been filtered for minimum frequency of 14 853, quality reads per sample and rarefication were adjusted to a sampling depth of 14 853 reads for further downstream analysis. Alpha (Shannon, evenness, Observed OTUs, Faith PD) and Beta diversity (Bray Curtis, Jaccard, weighted and unweighted UniFrac) were calculated to compare microbial communities using Qiime’s plugin “qiime diversity core-metrics-phylogenetic” and “qiime diversity beta-group-significance” to perform ANOSIM and PERMANOVA tests on beta diversity metrices and the Kruskal-Wallis to test for alpha diversity differences. Feature tables from Qiime2 were transformed according to the workflow of LEfSe into the appropriate format and for further differential abundance analysis transferred to LEfSe analysis within a Galaxy environment (Version 1.0).^16^

Plots were generated with R (version 3.6.2) in RStudio (1.1.463) using the packages tidyverse (1.3.0), qiime2r (0.99.6), ggplot2 (3.3.3), dplyr (1.0.6), and ggpubr (0.4.0.999), respectively.

The data sets generated and analyzed during the current study are available in the European nucleotide archive (ENA) repository under the primary accession number PRJEB48579 (secondary accession ERP132965).

#### Modelling of microbial community metabolism

Representative nucleotide sequences of the calculated OTUs were mapped to the 16S ribosomal RNA genes within genomes of the Human Reference Gut Microbiome (HRGM) catalogue,^17^ which consists of 5414 distinct prokaryotic species. Mapping was based on pairwise sequence alignments using *BLASTN* (version 2.9.0+)^18^ with a minimum query (OTU) coverage of 95% and a minimum sequence identity of 97%. In case of multiple hits, only hits with the maximum identity were retained for further analysis. Genome-scale metabolic models of individual genomes were reconstructed using *gapseq* (version 1.1 commit 56466f).^19^ All metabolic models and the exact *gapseq* commands for the reconstructions are available at Zenodo.^20^

In order to predict microbial community metabolic processes, the individual metabolic network models of bacteria found with a relative abundance of >0.1% in the respective sample were merged into multispecies models as described previously.^5,9^ In brief, based on the multispecies models, metabolic fluxes including metabolite production rates (ie, butyrate) were predicted using parsimonious flux balance analysis^21^ that maximizes the biomass formation rate by the community minus the total sum of absolute reaction fluxes scaled by a factor of 10^-5^. The butyrate production rate was calculated as the outflow of butyrate divided by the predicted production rate of the community biomass. The simulations were performed in R (version 4.1.2) in connection with the *sybil* package.^22^ All scripts for running the community metabolism simulation are made available in *github* (https://github.com/Waschina/Graz_Cortison).

## Results

### Patient Characteristics

Ninety-three patients were included in the analysis, of whom all provided fecal samples at V1, while fecal samples at V2 were available from 85 patients (Supp. Figure 1).

**Figure 1.**
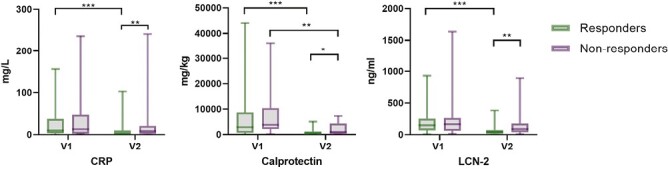
Serum and fecal biomarkers in responders (*n* = 69) compared with nonresponders (*n* = 24) at baseline (V1) and after 4 weeks of corticosteroid therapy (V2) displayed with boxplots. Significances are indicated with bars and stars within the diagrams. *P ≤ 0.05; **P ≤ .01; ***P ≤ .001. Green: responders, purple: nonresponders. LCN-2 = Lipocalin-2.

Of the included patients, 42 (45%) were females, the median age was 38 years, and median disease duration 5 years at study inclusion. Forty (43%) patients suffered from severe colitis (Lichtiger Score >10), and 34 (37%) patients were hospitalized due to the acute flare. Forty-three (46%) patients had already been exposed to corticosteroids (Table 1). Overall, 69 (74%) patients responded to systemic corticosteroids. Except for lower hemoglobin levels (*P* = .04) in nonresponders, patient characteristics of responders and nonresponders at V1 did not differ. At V2, nonresponders had lower hemoglobin (*P* = .04) and albumin levels (*P* = .001) but higher CRP (*P* = .01), calprotectin (*P* = .01), and LCN-2 (*P* = .005) concentrations (Supp. Table 1, Figure 1). Responders showed significant reductions of CRP, calprotectin, and LCN-2 from V1 to V2 (all *P* < .001). Nonresponders experienced no change in CRP and LCN-2 but also experienced a significant reduction of calprotectin (*P* = .005) from V1 to V2.

**Table 1.** Patient characteristics of the study cohort (*n* = 93) at baseline (V1). Data presented as median (q1, q3) or *n* (%).

Patients (*n* = 93)	V1
Age (years)	38 (29, 58)
Female sex, n (%)	42 (45)
Body-mass index	23.8 (21.7, 27.1)
Disease duration (years)	5 (1, 13)
Hemoglobin (g/dL)	13.6 (12.8, 14.6)
Leukocytes (10^9^/L)	7.9 (6.4, 10.0)
Thrombocytes (10^9^/L)	303 (252, 403)
C-reactive protein (mg/L)	10 (2, 31)
Albumin (g/dL)	4.1 (3.7, 4.5)
Fecal calprotectin (mg/kg)	3516 (996, 9255)
Fecal lipocalin-2 (ng/mL)	147 (64, 266)
Lichtiger Score	10 (9, 12)
Severe colitis, n (%)	40 (43)
Hospitalization, n (%)	34 (37)
**Disease extent/Montreal classification, *n* (%)**	
E1	10 (11)
E2	38 (41)
E3	44 (47)
Unknown	1
**Steroid dose (mg)**	
Prednisolone	50 (50, 50)
Methylprednisolone	40 (40, 64)
**Concomitant therapy, *n* (%)**	
Topical 5-ASA	24 (26)
Oral 5-ASA	69 (74)
Sulfasalazine	6 (7)
Topical budesonide	3 (3)
Azathioprine	7 (8)
Biologics	19 (20)
**Previous therapy, *n* (%)**	
Azathioprine	17 (18)
Corticosteroids	43 (46)
Biologics	15 (16)

### Microbiome Analysis

#### Response to corticosteroid therapy could not be predicted by distinct microbial composition prior to corticosteroid treatment

**Figure 2.**
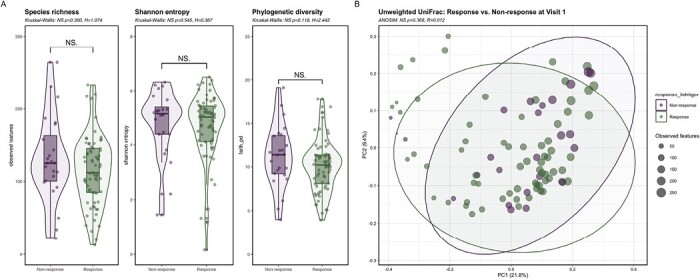
Comparison of alpha and beta diversity in responders vs. nonresponders to corticosteroids at baseline (V1). A, Alpha diversity showed no difference in responders compared with nonresponders at V1 determined by species richness, Shannon entropy and phylogenetic diversity (Kruskal Wallis: *P* > .05; response, *n* = 69; nonresponse, *n* = 24). B, Unweighted UniFrac showed no qualitative phylogenetic difference in the microbial composition between responders and nonresponders at V1 (ANOSIM: *P* > .05; response, *n* = 69; nonresponse, *n* = 24). Green: responders, purple: nonresponders. Abbreviation: NS, not significant.

We were not able to detect a microbiome signature at baseline predicting subsequent response to corticosteroid treatment after 4 weeks. Alpha and beta diversity between responders and nonresponders did not significantly differ at baseline (Figure 2), and differences in taxonomy could not be observed if choosing an LDA Score of ≥4 as cutoff by LEfSe analysis (Supp. Table 2).

#### Microbial composition longitudinally changed in responders during corticosteroid therapy

**Figure 3.**
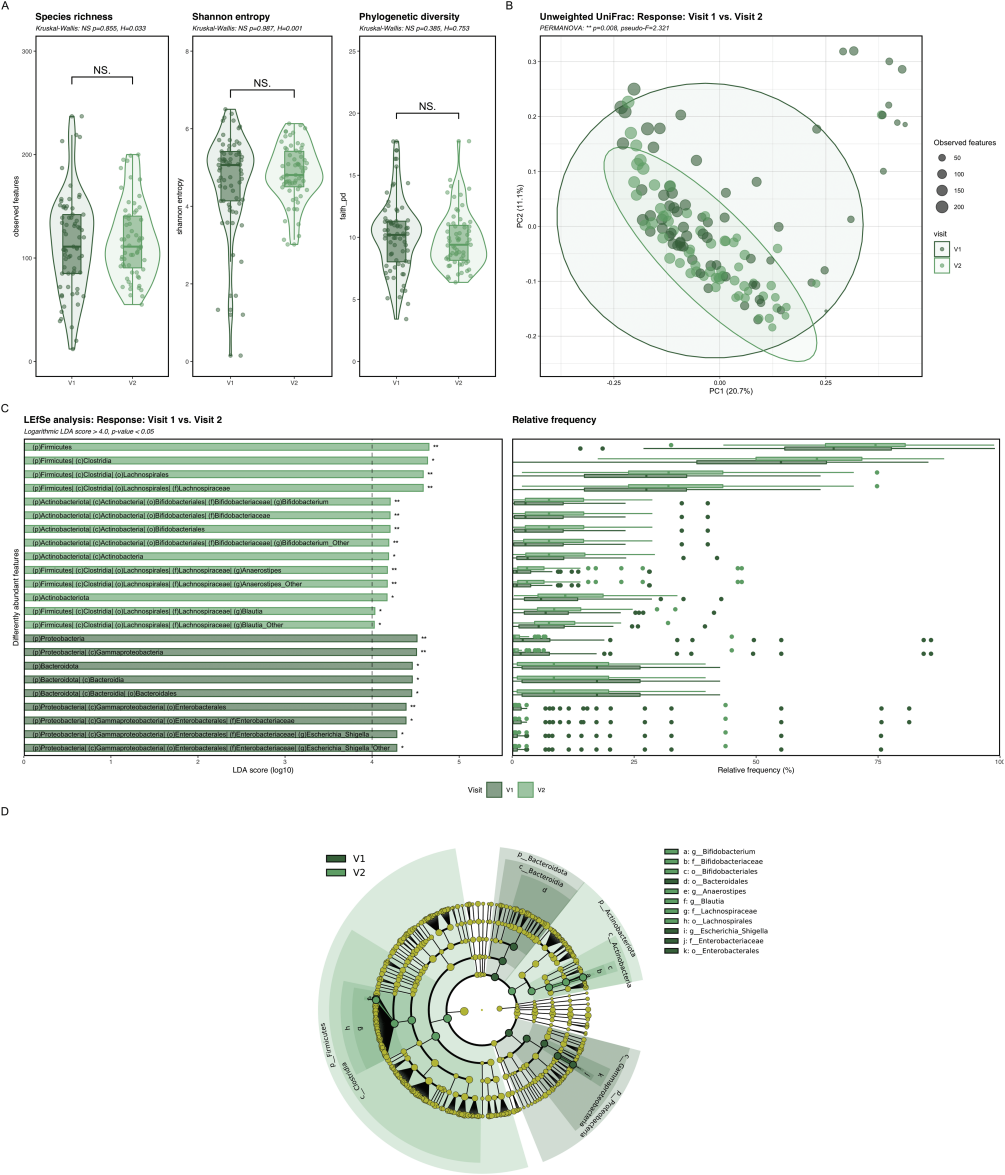
Changes in alpha and beta diversity and taxonomy in responders during the 4 weeks of corticosteroid therapy. A, Alpha diversity showed no significant changes in samples of responders at V1 compared with V2 determined by species richness, Shannon entropy and phylogenetic diversity (Kruskal Wallis: *P* > .05; V1 *n* = 69; V2 *n* = 63). B, Beta diversity showed a significant qualitative (Unweighted UniFrac) phylogenetic difference in the microbial composition of responders at V1 compared with V2 (PERMANOVA: *P* < .05; V1 *n* = 69; V2 *n* = 63). C, D, LEfSe analysis showed 23 significantly discriminative features in responders comparing microbial taxonomy in fecal samples at V1 and V2 with relative abundance (Logarithmic LDA score ≥4.0; P < .05; V1 *n* = 69; V2 *n* = 63). Dark green: baseline (V1), light green: after 4 weeks of corticosteroid therapy (V2). Abbreviation: NS, not significant. *P ≤ .05; **P ≤ .01.

Longitudinally, alpha diversity did not change from V1 to V2 in responders, but treatment changed quantitative (weighted UniFrac) and qualitative (unweighted UniFrac) microbial composition. With LEfSe analysis, 23 taxa discriminating V1 from V2 were identified with an LDA Score of ≥4 as cutoff (Supp. Table 2). V1 was associated with increased *Proteobacteria* and *Bacteriodotes* and the classes *Bacteroidia* and *Gammaproteobacteria* containing the genus *Escherichia Shigella*. *Firmicutes* and *Actinobacteriota* and the genera *Blautia*, *Anaerostipes*, and *Bifidobacterium* were increased at V2 (Figure 3).

#### Nonresponders had minor changes in microbial composition during corticosteroid treatment

**Figure 4.**
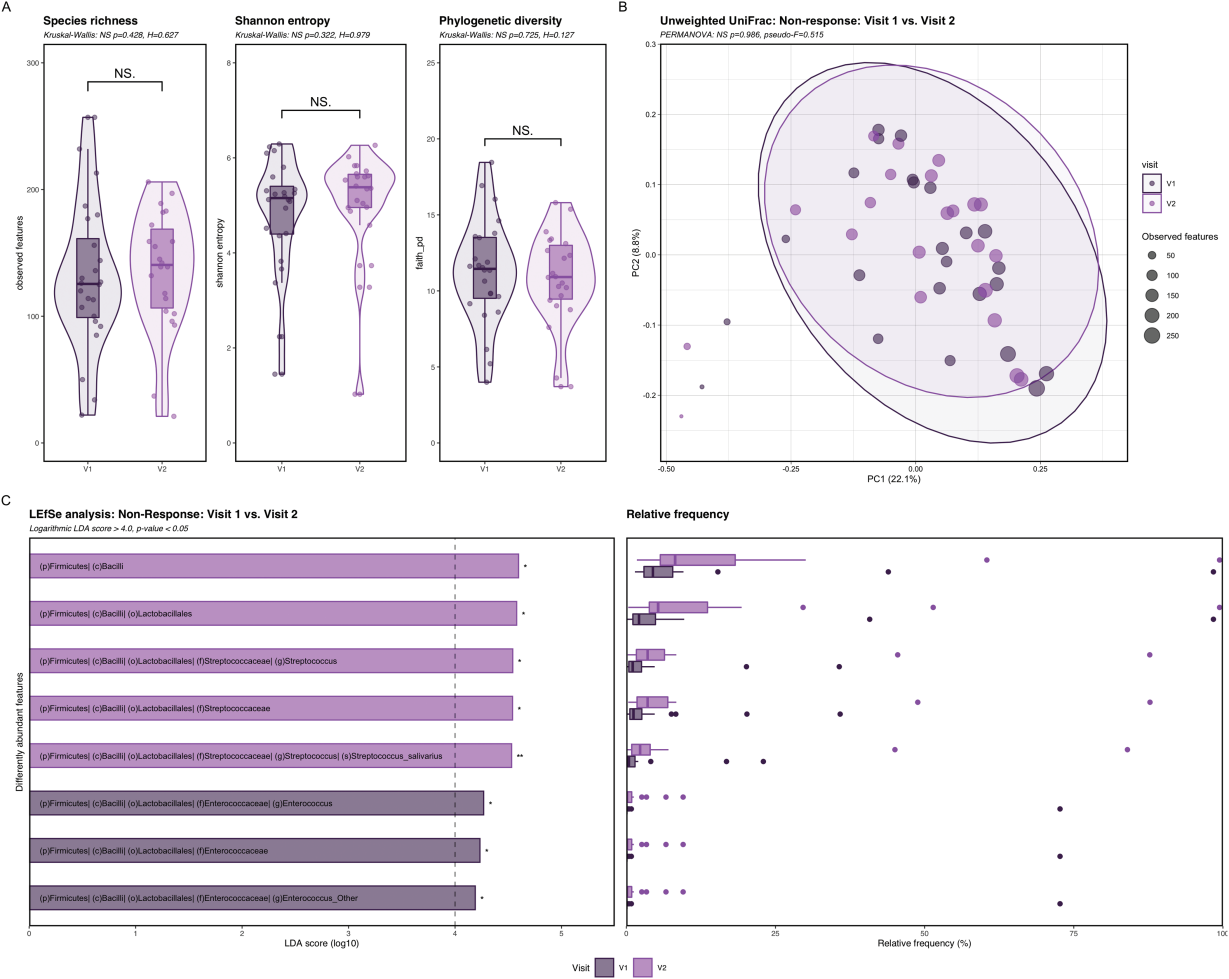
Changes in alpha and beta diversity and taxonomy in nonresponders during the 4 weeks of corticosteroid therapy. A, Alpha diversity showed no significant changes in samples of nonresponders at V1 compared with V2 determined by species richness, Shannon entropy and phylogenetic diversity (Kruskal Wallis: *P* > .05; V1 *n* = 24; V2 *n* = 22). B, Beta diversity showed no significant differences in the microbial composition of nonresponders at V1 compared with V2 as shown for unweighted UniFrac (PERMANOVA: *P* > .05; V1 *n* = 24; V2 *n* = 22). C, LEfSe analysis showed 8 significantly discriminative features in nonresponders comparing microbial taxonomy in fecal samples at V1 and V2 (Logarithmic LDA score ≥4.0; *P* < .05; V1 *n* = 24; V2 *n* = 22). Dark purple: baseline (V1), light purple: after 4 weeks of corticosteroid therapy (V2). Abbreviation: NS, not significant. *P ≤ .05; **P ≤ .01.

 Corticosteroids did not longitudinally change alpha and beta diversity in nonresponders from V1 to V2. Relative abundance of *Enterococcus* was significantly higher before treatment, whereas treatment increased relative abundance of *Streptococcus* with the species *S. salivarius*, a bacterium usually inhabiting the oral cavity (Figure 4).

#### Microbial composition was distinct in responders and nonresponders after 4 weeks of corticosteroid treatment

**Figure 5.**
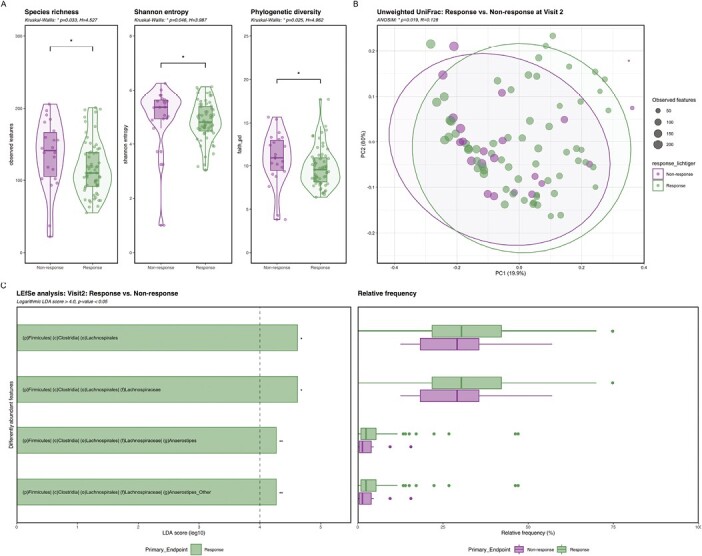
Comparison of alpha and beta diversity and taxonomy in responders vs nonresponders to corticosteroids at week 4 (V2). A, Alpha diversity was significantly reduced in responders compared with nonresponders after 4 weeks of corticosteroid treatment determined by species richness, Shannon entropy and phylogenetic diversity (Kruskal Wallis: *P* < .05; response *n* = 63; nonresponse *n* = 22). B, Unweighted UniFrac showed a significant qualitative phylogenetic difference in the microbial composition between responders and nonresponders at V2 (ANOSIM: *P* < .05; response *n* = 63; nonresponse *n* = 22). C, LEfSe analysis showed four significantly discriminative features at V2 that were associated with response (Logarithmic LDA score ≥4.0; P < .05; V1 *n* = 63; V2 *n* = 22). Green: responders, purple: nonresponders. *P ≤ .05; **P ≤ .01.

Treatment with corticosteroids led to discrimination of microbial composition between responders and nonresponders at V2. Responders had lower alpha diversity compared with nonresponders at V2 and distinct qualitative beta diversity using the unweighted UniFrac metrics. Response was further associated with increased abundance of the proposed butyrate-producing genus *Anaerostipes* (Figure 5).

#### Simulations of microbial community metabolism indicated increasing butyrate production capacity only in responders

**Figure 6.**
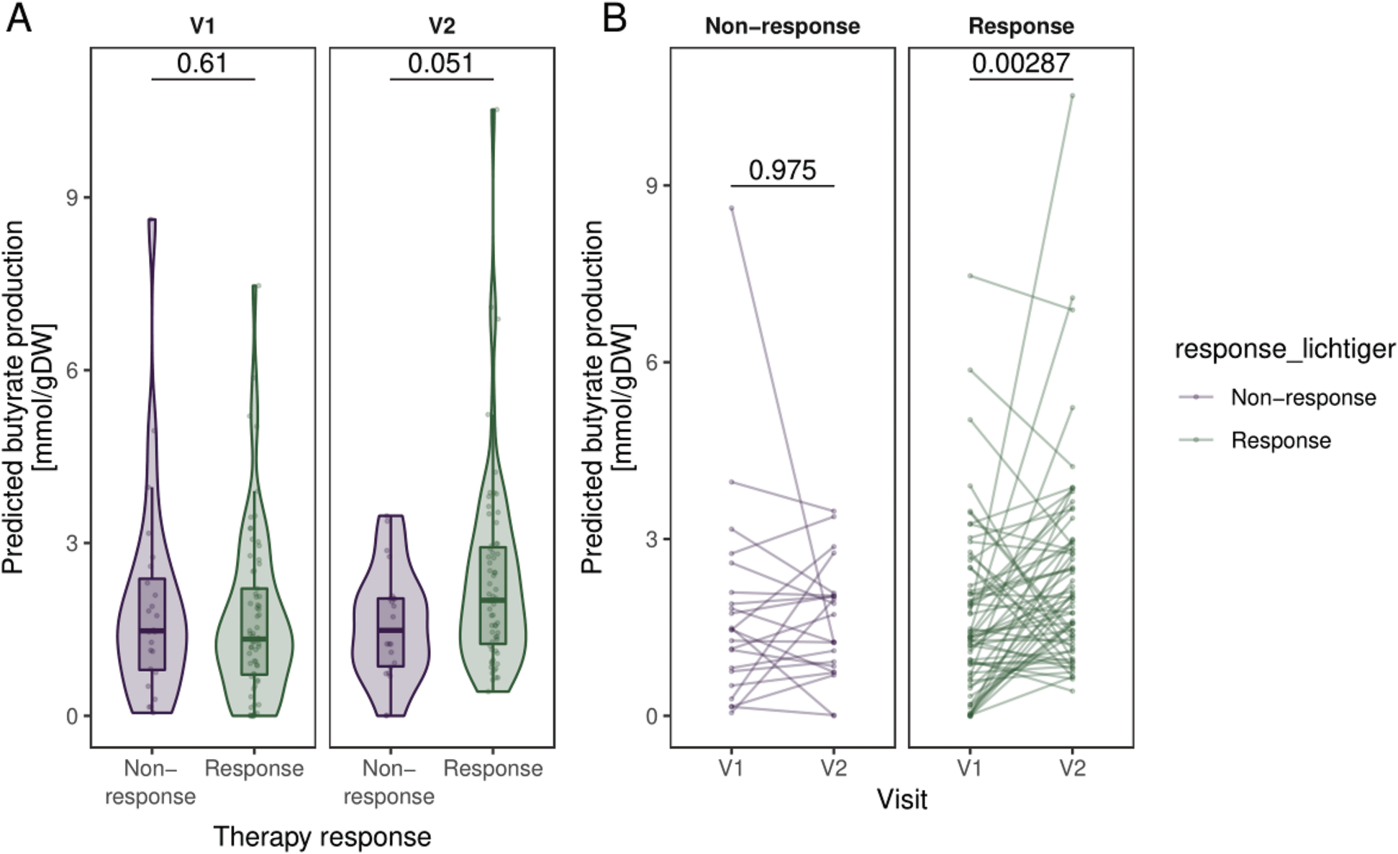
Predicted butyrate production by the gut microbial communities at baseline (V1) and after 4 weeks of corticosteroid therapy (V2) compared between responders and nonresponders and between time points. A, Comparison of butyrate production rate between responders and nonresponders at V1 and V2. *P* values are based on Wilcoxon rank sum exact test. B, Comparison between V1 and V2 stratified by therapy response to corticosteroids. Connected dots refer to the same patient. *P* values are calculated from the paired Wilcoxon signed rank exact test. V1 *n* = 93, V2 *n* = 85.

We used metabolic flux simulations to predict the butyrate production capacity of the sampled microbial communities. No significant differences in butyrate production rate were observed at baseline between responders and nonresponders (Wilcoxon rank sum exact test, *P* = .61). After 4 weeks of treatment, patients who responded to corticosteroids tended to have higher gut microbial butyrate production capacity compared with nonresponders (*P* = .051, Figure 6A). The longitudinal data further allowed us to perform paired statistical tests in order to check whether butyrate production rates changed during treatment. Although no change was detected among nonresponders (Wilcoxon signed rank exact test, *P* = .975, Figure 6B), responders showed a significantly increased predicted butyrate production (*P* = .003, Figure 6B). These results suggest that restoration of the gut microbial capacity to produce the short-chain fatty acid butyrate is associated with therapy response.

#### Response to corticosteroid therapy was associated with distinct microbial diversity and composition prior to corticosteroid treatment in biologic-naïve patients

In addition to the main analysis, we evaluated the existence of a microbiome signature at baseline discriminating later responders and nonresponders in subgroups. With microbial diversity and composition, we were not able to distinguish responders from nonresponders in the subgroups of corticosteroid-naïve, corticosteroid-preexposed, and biologic-exposed patients and in the subgroups of patients with and without concomitant oral aminosalicylate intake. In the subgroup of biologic-naïve patients, responders to corticosteroids had a significant lower baseline alpha diversity and a distinct qualitative (unweighted UniFrac) microbial composition compared to nonresponders. The order *Lactobacillales* was associated with response and the family *Oscillospiraceae* with nonresponse (Supp. Figure 2). Further, in the subgroups of patients with pancolitis and left-sided colitis, no difference in alpha and beta diversity between responders and nonresponders was detected at baseline.

## Discussion

This prospective, multicenter study shows that response to systemic corticosteroids in active UC is associated with taxonomic and functional restoration of the fecal microbiome, highlighted by the increase of beneficial genera and predicted butyrate production. Microbial diversity and composition before treatment differed between responders and nonresponders in the subgroup of biologic-naïve patients but showed no differences in the total cohort of patients.

The availability of an increasing number of advanced treatment options for UC including biologics, janus kinase inhibitors, and recently sphingosine-1-phosphate inhibitors^2,23^ increases the need to identify patients suitable for certain drugs, preferably prior to treatment initiation to avoid unnecessary drug exposures and potential side effects. So far, no biomarker has been identified reliably predicting treatment response to approved medications in IBD, and therapeutic decisions are mainly based on clinical information including the history of previous therapies, disease behavior and location, and disease activity. Fecal calprotectin is the best validated noninvasive biomarker in IBD to monitor intestinal inflammation^24^ and was able to predict mucosal healing early in the treatment course of biologics,^25^ as well as response to intravenous corticosteroids in a pediatric cohort.^26^ Modern approaches trying to predict response to treatment to biologics include multimodal -omics technologies with deep molecular profiling comprising transcriptomics, proteomics, and metabolomics.^27^ Microbial composition as a prediction tool for treatment outcome of biologics in IBD has already been investigated in several studies. Increased alpha diversity and higher abundance of the taxa *Roseburia* and *Burkholderiales* was observed in the feces of UC and Crohn’s disease patients subsequently achieving remission to vedolizumab therapy.^7^ In Crohn’s disease patients treated with ustekinumab, *Faecalibacterium* and *Bacteroides* were elevated in baseline fecal samples of patients with remission 6 weeks after treatment initiation.^8^ Both studies demonstrated that predictive modelling algorithms using microbial metadata from baseline samples can predict remission to therapy and seem to surpass clinical metadata. Elevation of *F. prausnitzii* in baseline mucosal biopsies of UC patients and altered expression of antimicrobial peptides were observed in responders to anti-TNFs.^6^ Aden et al were able to differentiate IBD patients achieving remission to anti-TNFs from patients without remission by fecal metabolites before treatment.^9^

We were not able to identify differences in microbial diversity, composition, or metabolic capacity at baseline between later responders and nonresponders to systemic corticosteroids in the main analysis of the total cohort. Except for hemoglobin levels, clinical and biochemical parameters did not significantly differ between the 2 groups before treatment, suggesting that clinical nonresponse to corticosteroids may not solely be explained by higher baseline inflammatory burden. Perhaps the lack of differing inflammatory burden between the groups, which is a relevant contributor to the microbial composition in IBD patients,^28^ may at least, in part, explain the lack of baseline differences.

When interpreting our data, one has to take into account the different concomitant medications administered during the study. As indicated by previous studies, biologics may have a relevant effect on the microbial composition.^8,9,29^ Therefore, a subgroup analysis of biologic-naïve patients was conducted, and baseline microbial diversity and composition were found to be associated with the later treatment outcome. Unexpectedly, baseline alpha diversity was higher in later nonresponders, a finding contrary to findings reported previously for biologics.^7,8^ Another unexpected finding is the association of the family *Oscillospiraceae* with nonresponse inasmuch as the beneficial genus *Faecalibacterium* belongs to this family and has been associated with favorable outcomes.^8,9^ It needs to be stated that the number of patients available for this subgroup analysis was limited and unbalanced; therefore, the results need to be interpreted with caution. In general, the composition of the fecal microbiome does not seem to be of major importance for the efficacy of systemic corticosteroids. The already established mechanisms of steroid resistance such as the defective glucocorticoid receptor binding and translocation and other responsible intracellular and signaling factors^30^ may therefore be of greater clinical importance for this phenomenon in UC patients.

Although our study did not have a control group of healthy subjects, differences in the fecal microbiome between UC patients and healthy controls are well established. Compared with healthy individuals, UC patients have lower alpha diversity, higher *Proteobacteria*, and lower *Firmicutes* and *Bacteroidetes* levels and higher abundance of the genus *Enterococcus*.^31,32^ In our cohort, microbial composition longitudinally changed during treatment in responders, whereas changes in nonresponders were only minor. The observed changes in responders with the decrease of the phylum *Proteobacteria* and the increase of *Firmicutes* during treatment indicate partial restoration of microbiome composition, as these changes lead to a microbial composition more similar to healthy individuals.^33,34^ The decrease of *Proteobacteria* was also observed in Crohn's disease patients responding to azathioprine or anti-TNF therapy in an Austrian cohort.^5^ We further observed an increase of *Actinobacteria* in responders after 4 weeks, a finding consistent with the elevation of the genus *Actinomyces* from the alike phylum in children from a pediatric UC cohort achieving remission with corticosteroids 4 weeks after treatment induction.^28^

Short-chain fatty acids, especially butyrate, are important metabolites of the gut microbiome and serve as energy sources and are involved in the maintenance of the intestinal barrier.^35^ We observed the increase of the butyrate-producing genera *Anaerostipes* and *Blautia*,^36,37^ and metabolic modelling of microbial communities revealed an increase in butyrate production capacity in responders. This indicates that the increase of microbial butyrate production is a central feature accompanied by response to therapy. The decrease of butyrate-producing species is a known feature of microbial alteration in UC,^38^ and increased butyrate synthesis has been linked to remission in IBD patients in other studies.^5^ Butyrate also suppressed dextran sulfate sodium colitis in mice and inhibited neutrophils of IBD patients to produce pro-inflammatory cytokines.^39,40^ Additionally, the beneficial genus *Bifidobacterium,* also containing short-chain fatty acid–producing species, increased in abundance in responders. *Bifidobacteria* had previously been described to be involved in immune homeostasis and the inhibition of adhesion of pathogens to the gut epithelium.^37^

The longitudinal increase of the oral commensal *Streptococcus salivarius* in nonresponders in this study was also observed in a previous investigation in UC patients not responding to vedolizumab treatment.^7^ This “oralization” of the fecal microbiome has also been linked to severe disease in pediatric, treatment-naïve UC patients.^28^

At week 4, the microbial composition differed between responders and nonresponders, and response was once again associated with the genus *Anaerostipes.* Unexpectedly, alpha diversity was lower in responders, a fact caused by a not significant longitudinal drop of diversity in responders and a slight increase in nonresponders. Possible explanations include an overgrowth of pathobionts in nonresponders or a restricted proliferation of new taxa in responders. Our data indicate that alpha diversity alone is probably not a suited parameter to measure microbiome restoration in IBD.

The limitations of this work comprise the heterogeneity of baseline inflammation, steroid administrations and dosing regimens, varying concomitant therapies, and the lack of endoscopic data. The use of the clinical Lichtiger score to define response to corticosteroids can also be interpreted as a weakness because it may lack sufficient accuracy to represent the real inflammatory burden in the colon. Further, 16S rRNA sequencing provides less taxonomy resolution than shotgun metagenomic sequencing techniques and does not allow functional profiling. An important strength of this study is the prospective, multicenter design in a real-world patient cohort, making results robust to selection bias by restrictive inclusion criteria as in phase 2/3 trials.

## Conclusion

Baseline microbial diversity and composition was associated with response to systemic corticosteroids in patients with active UC only in the subgroup of biologic-naïve patients, suggesting a limited influence of the microbial composition on corticosteroid efficacy. Response is longitudinally accompanied by microbial restoration and predicted butyrate synthesis.

## Supplementary Material

izad126_suppl_Supplementary_Material

## Data Availability

The data sets used and analyzed during the current study are available from the corresponding author upon reasonable request.
