# Supplementary material for: Prediction of Response to Systemic Corticosteroids in Active UC by Microbial Composition—A Prospective Multicenter Study
Source: Inflamm Bowel Dis. 2023 Jul 18;30(1):9–19. doi: 10.1093/ibd/izad126 (PMC10769779; doi:10.1093/ibd/izad126)
Supplement: izad126_suppl_Supplementary_Material [file izad126_suppl_supplementary_material.docx]

Supplement:


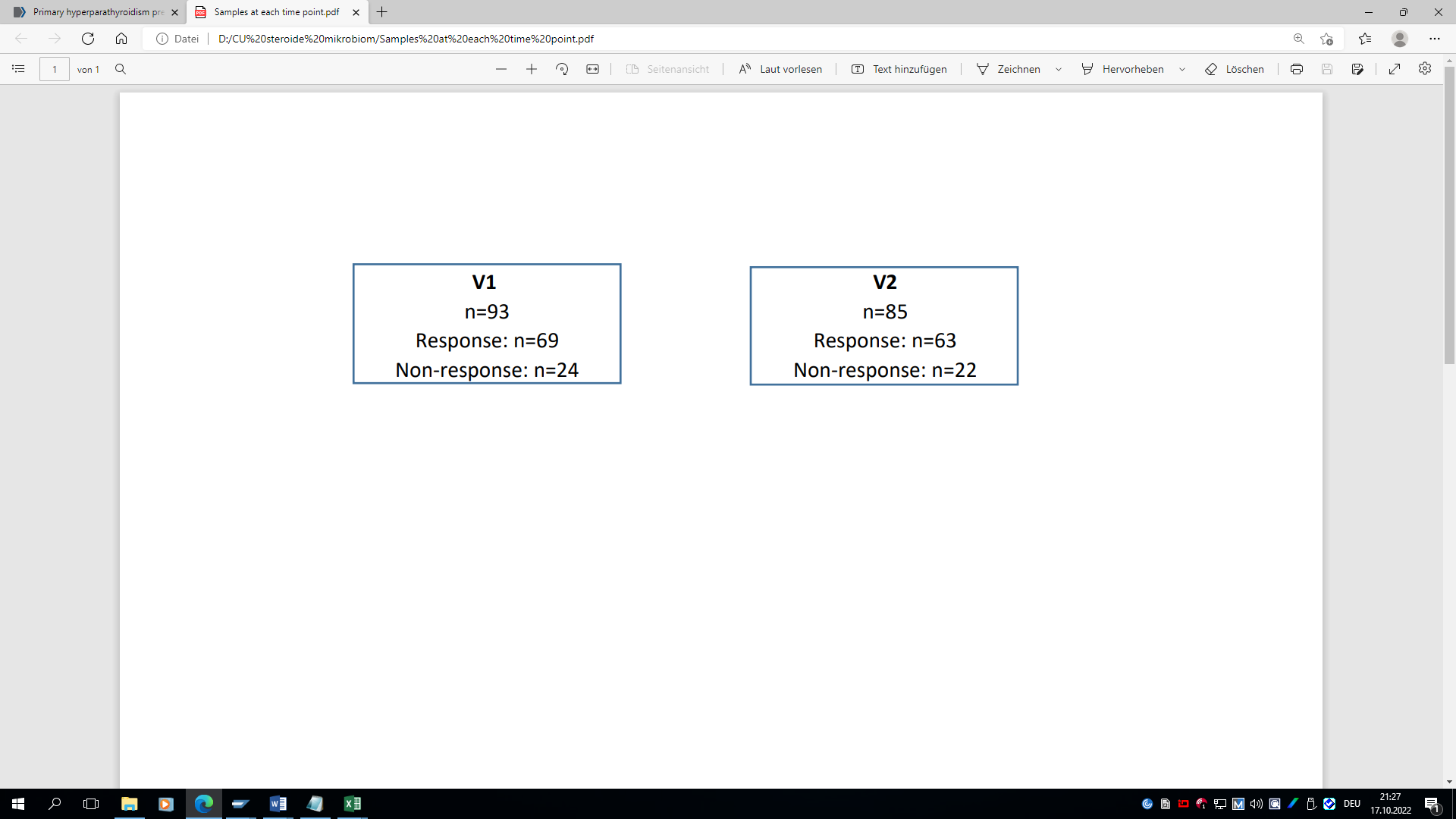


**Supp. Figure 1.** Included fecal samples in the analysis at baseline (V1) and after 4 weeks of corticosteroid therapy (V2) according to response and non-response to therapy. 113 patients have been screened for the study. Two patients were screening failures. 12 patients had to be excluded from the analysis due to missing fecal samples or missing clinical data to assess response to corticosteroid treatment. Further six patients had to be excluded due to initiation of concomitant biologics between V1 and V2.

**Supp. Table 1. Patient characteristics of responders (n=69) and non-responders (n=24) at V1 and V2**. Data presented as median (q1, q3) or n (%). P-values calculated with Chi square test or Mann-Whitney-U-Test as appropriate.

|  | **V1** | | | **V2** | | |
| --- | --- | --- | --- | --- | --- | --- |
|  | **Responders (n=69)** | **Non-Responders (n=24)** | **P-value** | **Responders (n=69)** | **Non-Responders (n=24)** | **P-value** |
| Age (years) | 38 (27, 58) | 39 (32, 58) | 0.3 |  |  |  |
| Female sex | 31 (45) | 11 (46) | 0.8 |  |  |  |
| Weight (kg) | 72 (64, 81) | 75 (66, 86) | 0.5 | 74 (64, 82) | 72 (61, 80) | 0.6 |
| Disease duration (years) | 3 (1, 11) | 9 (2, 17) | 0.1 |  |  |  |
| Hemoglobin (g/dL) | 13.7 (13.0, 14.7) | 13.3 (12.0, 13.8) | 0.04 | 13.4 (12.4, 14.6) | 12.7 (11.4, 13.7) | 0.04 |
| Leukocytes (10^9^/L) | 7.9 (6.6, 10.2) | 7.2 (6.0, 9.6) | 0.3 | 9.3 (7.4, 10.9) | 8.9 (7.9, 10.5) | 0.9 |
| Thrombocytes (10^9^/L) | 304 (254, 367) | 286 (244, 476) | 1.0 | 282 (235, 335) | 297 (241, 351) | 0.8 |
| C-reactive protein (mg/L) | 10.2 (2.9, 37.1) | 13.0 (2.0, 47.5) | 0.7 | 1.3 (0.6, 9.7) | 8.5 (2.2, 20.8) | 0.01 |
| Albumin (g/dL) | 4.1 (3.8, 4.5) | 3.9 (3.5, 4.5) | 0.3 | 4.3 (4.1, 4.5) | 3.9 (3.5, 4.1) | 0.001 |
| Calprotectin (mg/kg) | 2899 (727, 8744) | 3819 (2186, 10401) | 0.3 | 433 (68, 1225) | 1061 (236, 4350) | 0.01 |
| Lipocalin-2 (ng/ml) | 147 (69, 254) | 168 (62, 265) | 0.8 | 42 (16, 71) | 89 (39, 178) | 0.005 |
| Lichtiger Score | 10 (9, 13) | 10 (9, 12) | 0.4 | 2 (1, 3) | 8 (7, 9) | <0.001 |

**Supp. Table 2.** Discriminative features between groups and time points based on the relative abundance table from phylum to species level in LEfSe. Different time points and groups were compared to each other (first 2 columns) and discriminative features were identified (shown are absolute numbers of discriminative features, column 3). After internal Wilcoxon, number of significant features was reduced and further decreased if choosing higher LDA scores (column 4).

| Groups and  time points | Groups and  time points | Number of significantly discriminative  features before internal wilcoxon | Number of significantly discriminative  features with absolute LDA score > | | | | | |
| --- | --- | --- | --- | --- | --- | --- | --- | --- |
|  |  |  | 2 | 3 | 4 | 4.5 | 4.6 | 4.7 |
| V1 | R vs NR | 64 | 52 | 9 | 0 | 0 | 0 | 0 |
| V2 | R vs NR | 71 | 68 | 16 | 4 | 2 | 2 | 0 |
| Response | V1 vs V2 | 82 | 79 | 51 | 23 | 6 | 0 | 0 |
| Non-response | V1 vs V2 | 21 | 21 | 17 | 8 | 5 | 0 | 0 |

R=Response, NR=Non-Response, LDA=Linear discriminant analysis, V1: baseline, V2: week 4

**
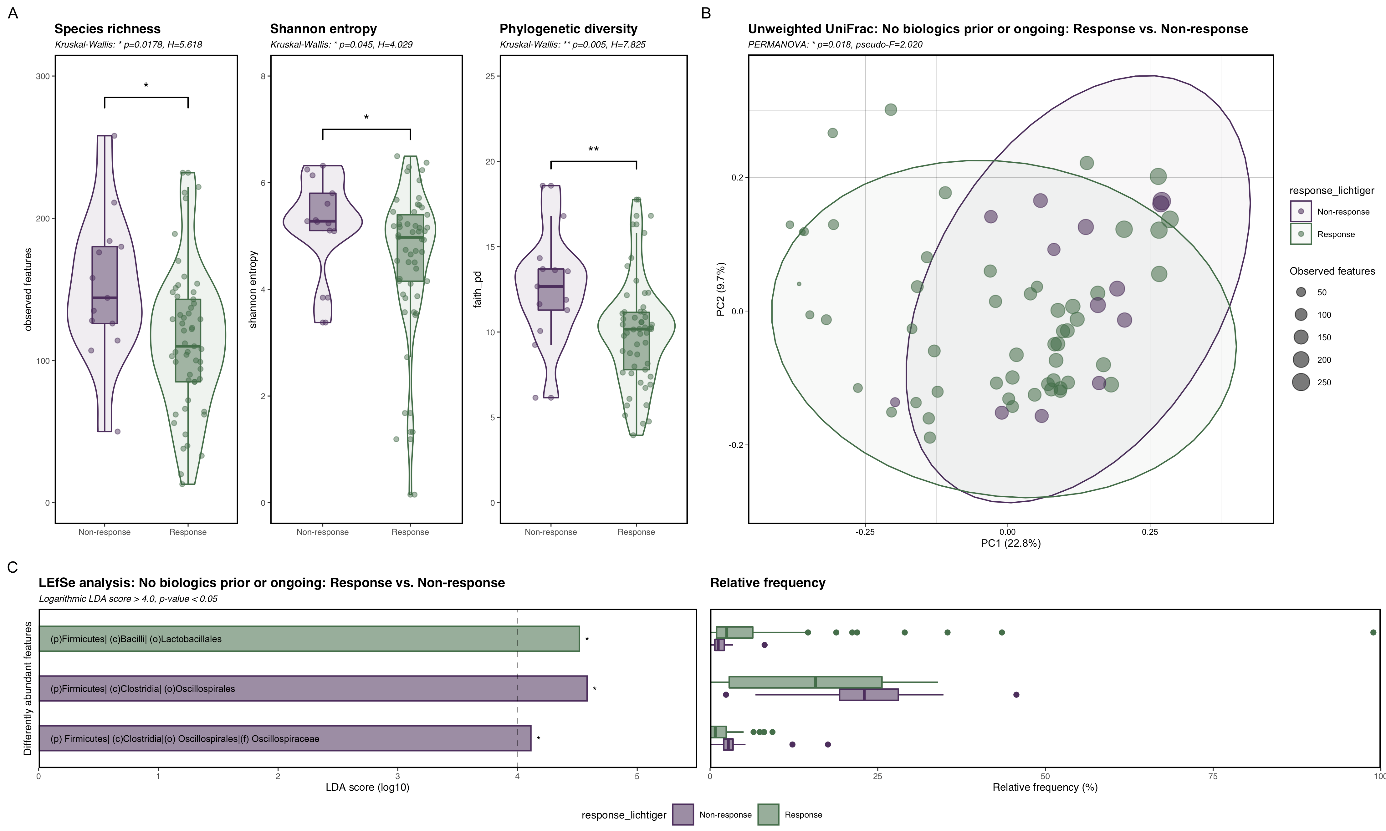
**

**Supp. Figure 2. Comparison of alpha and beta diversity and taxonomy in responders vs. non-responders to corticosteroids at baseline (V1)** **in biologic naïve patients (n=66).** **A**: Alpha diversity showed a significant increase of species richness, Shannon entropy and phylogenetic diversity in non-responders (Kruskal Wallis: p<0.05), Evenness was not significant different (data not shown). **B**: Unweighted UniFrac showed a significant qualitative phylogenetic difference in the microbial composition between responders and non-responders (PERMANOVA: p<0.05). **C**: LEfSe analysis showed a significant increase of *Lactobacillales* in responders, while *Oscillospirales* are significantly enriched in non-responders, both orders belong to the phylum *Firmicutes* (Logarithmic LDA score ≥ 4.0; p<0.05, response (n=53), non-response (n=13)). Green: responders, purple: non-responders. *p<0.05, **p<0.01, NS=not significant
